# Breastfeeding Apps: A Descriptive Report

**DOI:** 10.3390/bs13100801

**Published:** 2023-09-27

**Authors:** Silvia Cimino, Luca Cerniglia

**Affiliations:** 1 Department of Dynamic, Clinical and Health Psychology, Sapienza University of Rome, 00185 Rome, Italy; silvia.cimino@uniroma1.it; 2 Faculty of Psychology, International Telematic University Uninettuno, Corso Vittorio Emanuele II, 39, 00186 Rome, Italy

**Keywords:** breastfeeding apps, feeding interactions, psychopathological risk, motherhood

## Abstract

Background: Women are increasingly using breastfeeding apps to facilitate and organize breastfeeding; however, no study has so far focused on maternal psychopathological risk and on the quality of dyadic exchanges in this field. Aim and Methods: This preliminary, descriptive study aimed at evaluating levels of psychopathological symptoms (through the SCL-90/R) and for the quality of the interactions they have with their children during feeding (through the SVIA) in mothers who use breastfeeding apps with different grades of engagement. Results: Data analyses showed that mothers with a mild use of the apps present a higher quality of dyadic interactions during feeding and lower psychopathological risk. Conclusions: The cross-sectional and descriptive nature of this study does not allow any causal conclusions. However, results suggest that the higher the engagement and use of breastfeeding apps, the lower the quality of feeding interactions and the higher the maternal psychopathological risk.

## 1. Introduction

The trend among women to seek breastfeeding-related support from online platforms is on the rise [1,2,3,4]. In the context of this paper, the term “breastfeeding apps” is employed to encompass tools that facilitate and/or organize both breastfeeding and bottle feeding and that are used by mothers before, during and after the feeding. Notably, mobile phone-based sources, often categorized as mobile health (mHealth), have emerged as a particularly popular avenue. These encompass social media platforms and mobile apps [5]. Illustratively, an investigation conducted in 2016 among mothers in Australia revealed that a significant proportion, approximately three-quarters, had engaged with at least one app during the course of pregnancy. Furthermore, nearly half of the surveyed mothers acknowledged employing at least one app in the post-partum phase [6].

In general, the use of mobile apps to support parenthood is increasingly common, with many new parents turning to their smartphones for information, advice, and tools to help them in their new role [7,8,9,10]. While some studies have found that pregnancy and parenting apps can be useful sources of information and reassurance for new parents, others argue that many apps reinforce traditional gender roles, focus on mothers, and lack evidence-based content [11].

Within the framework of infant research articulated by Spitz and Emde [12] as well as Stern [13], it is posited that the infant possesses an inherent capacity for engagements that involve bidirectional communication between subjects. This early competence is prominently exhibited in the interactional dynamics between the infant and primary caregivers across varying contexts, such as feeding and play [14,15]. Stern [16] advances the notion that dyads instinctively regulate their bidirectional communications in divergent manners contingent upon the specific context and their respective intentions. During play-oriented interactions, the caregiver and the child are inclined to mutually engage, with a focus on reciprocal amusement [17]. In contrast, during feeding exchanges, the caregiver’s inclination is to directly guide the child’s conduct [18,19], thereby directing their attention towards the task at hand. In the natural progression of interactive exchanges, whether encompassing play or feeding, the dyad continuously engages in bidirectional adjustments [20]. This evokes an analogy proposed by Stern and Bruschweiler-Stern [21,22] likening the mother’s role to that of an orchestral conductor—a role that may also be attributed to the infant, or one that shifts reciprocally between them.

Literature in this field [16] underscores the significance of examining the contextual dimensions of both play and feeding to discern the distinctive attributes characterizing the parent–infant relationship [23]. However, alternate viewpoints posit a distinct emphasis on the feeding context. Specifically, if the dyadic relationship is unable to effectively synchronize and partake in sensitive interactions during feeding, there is a potential hindrance to the infant’s acquisition of affective self-regulation skills [24,25,26]. This deficit in mutual attunement could subsequently manifest in maladaptive emotional and behavioral symptoms over an extended period [27,28,29].

Several studies analyzed the content and features of various parenting and breastfeeding apps. Virani [30] reviewed several apps and found most provided helpful information and tools for new parents, though some lacked evidence-based content [31]. Moreover, the research on parenting apps reveals significant risks around privacy, data security and the spread of misinformation. Parents are increasingly relying on apps to track their child’s development, but many apps fail to protect users’ sensitive data or provide evidence-based information [32,33].

Some apps provide contradictory or misleading advice on controversial topics without citing medical sources [34]. For example, Womack and colleagues [35] found that only 50% of 48 pregnancy apps cited sources for their health recommendations, and many gave conflicting advice on issues such as alcohol use during pregnancy. This spread of misinformation could negatively impact maternal and child health.

Apart from the quality of the breastfeeding-related apps mothers use, two further key aspects to consider are how much time they spent using them and what features of the apps are in fact used by the mothers [36]. Some authors have posited that specific types of users can be identified among mothers [37,38,39]. The “straightforward basic trackers” category predominantly adheres to fundamental self-tracking practices across various modes. These individuals exhibit a tendency to record only a limited set of parameters, primarily encompassing temporal aspects such as breastfeeding duration. Moreover, their engagement with self-tracking activities is intermittent compared to other user types. Notably, this group predominantly refrains from delving into personalized data management functionalities and remains hesitant to share their collected data either within the app or on social media platforms.

Conversely, the “meticulous data collectors” exhibit a more intricate and systematic approach to their self-tracking endeavors. These individuals maintain a consistent regimen of data collection, characterized by a greater degree of granularity. In terms of interpersonal communication, this group is analogous to the “straightforward basic trackers”, with the only discernible variation being a slightly elevated engagement in conversational interactions.

The “advisory-oriented self-trackers” constitute a smaller cohort, marked by a heightened propensity for diverse forms of self-tracking. While their data collection practices may not be as exhaustive as those of the meticulous data collectors, this group actively accumulates a wide array of data types, albeit intermittently. It is worth noting that this segment is more inclined to incorporate wearable technology for breastfeeding purposes. Additionally, this category exhibits a pronounced inclination toward algorithmic feedback, encompassing personalized recommendations and incentives.

Furthermore, this cohort’s communicative behavior aligns with their advisory-focused proclivities. These users exhibit a pronounced proclivity to engage in face-to-face discussions concerning their self-tracking outcomes. In contrast to the other two groups, the advisory-oriented self-trackers are more predisposed to sharing their tracked data within the app or on social media platforms, indicating their enthusiasm for collaborative and advisory aspects of self-tracking systems. Previous research has shown that these three groups of mothers do not significantly differ for perceived parental distress and well-being, but they present poorer self-esteem and body appreciation [39]. However, no study to our best knowledge have so far evaluated whether mothers in the three groups significantly differ for levels of psychopathological symptoms and for the quality of the interactions they have with their children during feeding. Consistently with Chatoor’s model [40], the present study aims to fill this gap. Chatoor proposed a developmental classification (Zero-to-Three: 0–3, now revised and replaced by the DC: 0–5 system). Although this diagnostic proposal has now been updated, it is considered important to emphasize its aspects of utility, especially in clinical practice, which can still guide the work of professionals internationally. Unlike most nosologies, this takes into account not only the characteristics of the mother–child relationship but also the maternal psychopathology. Above all, Chatoor’s nosography allows for the evaluation of the child in different developmental phases, interpreted in light of the results of Infant Research, considering that, in the initial phase, the developmental process is characterized by a mechanism of regulation of physiological and emotional states. Subsequently, it is oriented by the quality of the attachment and reciprocity bond established with the caregiver, and finally (when the child is older) by a process of separation-individuation. One of the main aims of these apps in the specific realm of breastfeeding is to reassure the mother that she is doing well with the baby. They can propose apparently effective caregiving strategies (for feeding, sleep, etc.) to avoid or reduce possible moments of conflict and crisis between parents and offspring. Therefore, besides the objectives cited above, we aimed to focus specifically on the levels of conflict during breastfeeding according to the clinical and theoretical model of Chatoor [40]. This is a preliminary report presenting the first descriptive results elaborated on the gathered data.

## 2. Materials and Methods

### 2.1. Participants

A convenience sample of one hundred and seventy-eight dyads (N = 178 mothers and their children) was recruited with to the collaboration of centers for delivery preparation, which allowed contacts with mothers who followed the course and were in touch for follow up visits. The children (87 girls, 91 boys) were 3 months old (SD = 0.7), and their mothers’ mean ages were 32.1 (SD = 1.8). Ninety-two per cent of the children were firstborn, and all of them were been breast-fed in the period of this study. Mothers and children belonged to intact families for the 93% of cases and had to middle-class socioeconomic status (SES; [41]; ~25,000 euros per year) (Table 1). All mothers were Caucasian. All mothers included in this study were involved in caregiving practices with their children on a daily basis [average time spent weekly by the mothers with their children: 81.2 h (SD = 2.6) and signed an informed consent according to the Declaration of Helsinki. This study was authorized before its start by the Ethics Committee (N.6_6/22).

The criteria employed for the selection of participants encompassed the following factors: (a) the age of the children = 3 months, (b) the absence of documented mental or physical conditions in both the mothers and children, and (c) confirmation from mothers of consistent routine feeding for their children. A panel of proficient psychologists engaged with the sampled individuals. These psychologists administered a specially designed questionnaire, crafted in accordance with the methodology outlined by Lomborg et al. [42], and following Karnowski and Reifegerste’s work [43] to measure their engagement with self-tracking apps for breastfeeding. Based on the information reported in this tool, mothers were divided in groups. It is important to note that the division in groups was made based on the self-report description mother have been asked to produce through the Karnowski and Reifegerste’s questionnaire, not based on what observed during the assessment of mother–child feeding interactions. In fact, during feeding, mothers could or could not use the apps (no indication was given). As only one mother showed the advisory-oriented profile, this group was joint with the meticulous data collectors one (composed of N = 86 mothers) and analyses were conducted confronting advisory-oriented/meticulous collectors (AO-MC) group with the straightforward basic trackers (SBT; composed of N = 91 mothers) group (see the introduction section for a brief description of the characteristics of these groups). The dyads under investigation were subjected to a home-based observation, conducted through 20 min video-recordings during the midday lunch session. The feeding interactions documented were integral components of a regular meal. Trained psychologists, specifically equipped with expertise in utilizing this observational tool, were responsible for recording the videos. Subsequently, two separate trained independent raters were engaged to analyze the videos. These raters closely followed the guidelines provided in the manual [44] while employing both a conventional paper-and-pencil system and a dedicated coding software program. The latter program was designed to facilitate the computation of scores on distinct subscales, including the Feeding Scale and the Observational Scale for mother-infant interaction during feeding [40,41,42,43,44], as outlined in prior works [45,46,47,48,49,50,51,52,53,54]. Moreover, mothers filled out the Symptom Checklist-90-Revised (SCL-90-R; [55]) for the self-report assessment of psychopathological symptoms.

### 2.2. Measures

The evaluation of the psychological well-being of mothers was conducted using the SCL-90-R. This self-report symptom inventory, devised by Derogatis [55], comprises 90 items and aims to quantify psychological symptoms and overall psychological distress. The assessment involves assigning scores to and interpreting results across nine primary subscales: Somatization, Obsessive-Compulsivity, Interpersonal Sensitivity, Depression, Anxiety, Hostility, Phobic Anxiety, Paranoid Ideation, and Psychoticism. Additionally, three Global Indices of Distress—namely the Global Severity Index, Positive Symptom Distress Index, and Positive Symptom Total—are utilized for interpretation. Responses on the SCL-90-R are measured using a Likert scale, with participants indicating their level of agreement on a scale from 0 (not at all) to 4 (extremely). Confirming previous studies [56,57,58,59] in this research, the SCL-90-R showed sound internal consistency (α = 0.81).

Evaluation of parent–child feeding interactions was carried out using the Italian version (SVIA—Feeding Scale) of the Observational Scale, as outlined by Chatoor et al. [40] and later adapted by Lucarelli et al. [44]. The scale encompasses 41 items, rated on a Likert scale from 0 to 3. It is organized into four subscales, analyzing dyadic interactions separately for both mother–child and father–child pairs. The subscales are as follows: Affective State of the Parent (e.g., parental display of sadness during feeding), Interactional Conflict (e.g., parental imposition of food into the child’s mouth), Food Refusal Behaviors of the Child (e.g., child’s refusal to open their mouth), and Affective State of the Dyad (e.g., manifestations of joy by both parent and child during feeding).

Higher scores in the Affective State of the Parent subscale signify increased difficulties in the caregiver’s ability to exhibit positive emotions, indicating a higher frequency of negative emotions such as sadness or distress. Interactional Conflict evaluates both the existence and intensity of conflictual exchanges within the dyad, highlighting instances where parental behavior during the meal is driven by their emotions and intentions, rather than being responsive to the child’s cues. The Food Refusal Behaviors of the Child subscale delves into behavioral and emotional attributes of the child’s feeding patterns, encompassing traits such as distractibility, opposition, or negativity.

Elevated scores on the Affective State of the Dyad subscale suggest challenges faced by the caregiver in supporting the child’s independent initiatives, often involving requests, authoritative directives, and critical behavior, while the child experiences distress and opposition.

The Italian version of this scale, validated for mother–infant interactions by Lucarelli et al. [44], demonstrated correct group classification rates ranging from 82% to 92% in discriminant analysis. Additionally, the tool’s construct validity was substantiated. Interrater reliability ranged between 0.82 and 0.92, as measured by intraclass correlation coefficients.

### 2.3. Data Analysis

The comparative analysis of scores for both the AO-MC and SBT groups across all assessments was conducted utilizing repeated measures analyses of variance (ANOVAs). The obtained *p* values are presented, with statistical significance being acknowledged for values below 0.05. The mean values, along with their corresponding standard deviations (SDs), are also reported. In accordance with Cohen’s guidelines [60], a power analysis was conducted to determine whether the sample size was adequate, establishing an α level of 0.05. The outcome yielded a power of 0.891, corresponding to a substantial effect size (f^2^ = 0.54).

## 3. Results

An analysis of variance (ANOVA) showed significant main effects related to group belonging (all *p* < 0.001) for all four subscale scores of the SVIA. Bonferroni’s post hoc tests indicated that SVIA scores in straightforward basic trackers group were notably lower (i.e., less maladaptive) than those of group advisory-oriented/meticulous data collectors across all four subscales: mother’s affective state, interactive conflict, food refusal, and dyad’s affective state. The average scores for each SVIA subscale for the two groups of mothers, along with the η^2^ values, are documented in Table 2.

Furthermore, an ANOVA assessing the SCL-90/R subscales and Global Severity Index scores for mothers in the two groups points revealed a noteworthy main effect (*p* < 0.001). GSI scores exhibited a significant higher in mothers of group AO-MC. Specifically, mothers exhibited higher scores in the subscales of Obsession-Compulsive; Depression and Anxiety. Moreover, mothers in group AO-MC showed significantly higher problems at the GSI subscale (Table 3).

## 4. Discussion

This preliminary, descriptive study aimed at evaluating levels of psychopathological symptoms and for the quality of the interactions they have with their children during feeding in mothers who use breastfeeding app with different grades of engagement.

Our results showed that the quality of feeding interactions between mothers who used apps with a mild level of engagement (straightforward basic trackers) and their children was higher than in dyads with mothers with higher engagement in the apps. That was true in all sub-dimensions of the SVIA, suggesting a general impoverishment of feeding exchanges when mothers were more active in the use of the apps. It must be noted that in this study we measured the use of apps before, during and after the feeding interaction. Therefore, it can be speculated that the quality of the exchanges in these dyads suffered from the shift of maternal attention from the “here and now” of the breastfeeding to the use of the app [50,51,52,53,54]. It has been posited that during feeding mothers and children share an empathic attunement in which the attention of both partners (especially the adult) is focused on the child, with the aim of getting to know and recognize his/her signals [14,43]. This reciprocal attention is key for the child to construct the experience of being and to structure effective strategies of emotional/behavioral regulation [55,56,57,58]. If the mother is not focused on the child’s cues, this could result in her poorer sensitivity and contingency, which have been widely associated with negative outcomes for the child in later life. Noteworthy, in this study we do not have specific data on maternal sensitivity and contingency, nor on offspring possible later symptoms. However, the SVIA taps the capacity of the dyads to share a positive emotional climate and to be mutually attuned, both emotionally and behaviorally. Thus, we can hypothesize that the use of breastfeeding app, if performed with the characteristics of advisory-oriented and meticulous data collectors is associated with lower attunement [59,60,61,62]. It is very important to notice that all mothers in this study (both in the SBT and in the AO-MC groups) showed non-clinically relevant scores at the SVIA. All the scores were below the threshold of clinical attention. However, differences between the groups were noted.

Our results also showed that mothers using the apps more intensely present more psychopathological symptoms. Specifically, they are more at risk for Obsession-Compulsive; Depression and Anxiety problems. Moreover, mothers in group AO-MC showed significantly higher problems at the global severity index subscale, indicating a general higher distress. As for the scores of the feeding interactions, in this case also no mother showed clinically relevant levels of symptoms. However, especially for the obsessive-compulsive problems mothers in the group AO-MC presented scores definable as at-risk according to previous research [43]. It is evident that the cross-sectional nature of this study does not allow any causal conclusions, so that it can be equally possible that mothers with higher obsessive-compulsive symptoms use the apps more intensely to control their discomfort for a complex and generally unpredictable situation as breastfeeding, or that the use of these apps is predictive of symptoms. The same reasoning can be applied to the depression and anxiety symptoms [63,64,65,66]. Further studies should clarify this link. The descriptive nature of the present study only allows us to photograph some characteristics of the sample associated with the use of breastfeeding apps. Notwithstanding this limitation, this study is the first, to our best knowledge to focus on the quality of feeding interactions and on maternal psychopathological risk in groups of mothers using breastfeeding apps.

## 5. Conclusions

In summary, this study serves as an initial exploration into the multifaceted dynamics surrounding the use of breastfeeding apps, mother–infant feeding interactions, and maternal psychopathological risk. It underscores the need for future research to delve deeper into the mechanisms and causal pathways underlying these relationships, as well as the potential long-term consequences for both maternal well-being and child development. While this study provides valuable insights, it represents only a snapshot of the characteristics associated with breastfeeding app usage, leaving room for further inquiry into this growing area of maternal and infant health.

## Figures and Tables

**Table 1:** Demographic characteristics of the subjects of this study.

	AO-MC	SBT	N_tot_
Children’s gender	41 M; 45 F	46 M; 45 F	356
Children’s age, M (SD)	3.2 (0.42)	3.15 (0.21)	
Mothers’ age, M (SD)	32.4 (1.51)	32.9 (1.42)	
Household income	Approx. 2500 euros/month	Approx. 2500 euros/month	
Educational level	At least 12 years of schooling	At least 12 years of schooling	

M = male; F = female.

**Table 2:** Average scores and standard deviations of the SVIA subscales and general quality of mother–child feeding interactions.

	SBT	AO-MC	
	M (SD)	M (SD)	η^2^
Mother’s affective state	4.3 (3.13)	11.1 (2.21) **	0.68
Interactive conflict	3.6 (3.61)	6.2 (3.32) **	0.56
Food refusal behavior	3.2 (2.17)	5.4 (1.48) **	0.62
Dyad’s affective state	2.9 (2.51)	4.3 (1.74) **	0.62

η^2^: eta-squared, ** *p* < 0.001.

**Table 3:** Maternal scores at SCL-90-R.

	SBT	AO-MC	
	M (SD)	M (SD)	η^2^
SOM	0.21 (0.45)	0.34 (0.48)	0.18
O-C	0.25 (0.58)	0.89 (0.52) **	0.67
I-S	0.21 (0.43)	0.37 (0.28)	0.11
DEP	0.15 (0.27)	0.78 (0.72) **	0.84
ANX	0.17 (0.51)	0.62 (0.65) **	0.92
HOS	0.21 (0.54)	0.30 (0.14)	0.12
PHOB	0.25 (0.63)	0.22 (0.32)	0.13
PAR	0.21 (0.41)	0.26 (0.34)	0.13
PSY	0.23 (0.52)	0.18 (0.23)	0.18
GSI	0.58 (0.42)	0.81 (0.76) **	0.65

*Note.* SOM: Somatization; O-C: Obsessive-Compulsive; I-S: Interpersonal Sensitivity; DEP: Depression; ANX: Anxiety; HOS: Hostility; PHOB: Phobic Anxiety; PAR: Paranoid Ideation; PSY: Psychoticism; GSI: Global Severity Index. η^2^: eta-squared, ** *p* < 0.001.

## Data Availability

Data are available through reasonable request to the corresponding author.
